# Cross-talk of membrane lipids and Alzheimer-related proteins

**DOI:** 10.1186/1750-1326-8-34

**Published:** 2013-10-22

**Authors:** Jochen Walter, Gerhild van Echten-Deckert

**Affiliations:** 1 Department of Neurology, University of Bonn, Sigmund-Freud-Str. 25, 53127, Bonn, Germany; 2 EURON–European Graduate School of Neuroscience, Bonn, Germany; 3 Life and Medical Sciences (LIMES), Membrane Biology and Lipid Biochemistry Unit at the Kekulé-Institute, University of Bonn, Gerhard-Domagk-Str. 1, 53121, Bonn, Germany

**Keywords:** Alzheimer’s disease, Sphingolipids, Gangliosides, Cholesterol, Tau, Beta-amyloid, Lysosomal storage disorders

## Abstract

Alzheimer’s disease (AD) is neuropathologically characterized by the combined occurrence of extracellular β-amyloid plaques and intracellular neurofibrillary tangles in the brain. While plaques contain aggregated forms of the amyloid β-peptide (Aβ), tangles are formed by fibrillar forms of the microtubule associated protein tau. All mutations identified so far to cause familial forms of early onset AD (FAD) are localized close to or within the Aβ domain of the amyloid precursor protein (APP) or in the presenilin proteins that are essential components of a protease complex involved in the generation of Aβ. Mutations in the tau gene are not associated with FAD, but can cause other forms of dementia. The genetics of FAD together with biochemical and cell biological data, led to the formulation of the amyloid hypothesis, stating that accumulation and aggregation of Aβ is the primary event in the pathogenesis of AD, while tau might mediate its toxicity and neurodegeneration.

The generation of Aβ involves sequential proteolytic cleavages of the amyloid precursor protein (APP) by enzymes called β-and γ-secretases. Notably, APP itself as well as the secretases are integral membrane proteins. Thus, it is very likely that membrane lipids are involved in the regulation of subcellular transport, activity, and metabolism of AD related proteins.

Indeed, several studies indicate that membrane lipids, including cholesterol and sphingolipids (SLs) affect Aβ generation and aggregation. Interestingly, APP and other AD associated proteins, including β-and γ-secretases can, in turn, influence lipid metabolic pathways. Here, we review the close connection of cellular lipid metabolism and AD associated proteins and discuss potential mechanisms that could contribute to initiation and progression of AD.

## Introduction

Alzheimer’s disease (AD) is the most common form of dementia, and defined at the neuropathological level by the presence of both extracellular plaques and intracellular tangles, associated with severe loss of synapses and neurodegeneration [1-3]. While neurofibrillary tangles (NFT) consist of paired helical filaments (PHF) of the microtubule-associated protein tau, amyloid plaques contain aggregated amyloid β-peptides (Aβ). Strong evidence from genetic, biochemical, and cell biological studies indicates a critical role of Aβ in the initiation of AD. All mutations that cause early onset forms of FAD affect the generation and/or aggregation property of Aβ, and are found either in the APP gene itself or in the presenilin (PS) genes [4,5]. As the respective PS proteins are the catalytic components of the γ-secretase complex, PS mutations are also directly linked to APP processing and commonly increase the relative abundance of the more aggregation prone Aβ42 variant as compared to Aβ40.

The mutations in the APP and PS genes are very rare and represent only 1-5% of all AD cases [4,6,7]. The causes of the much more common late onset forms of AD appear quite complex and likely involve age-related alterations in metabolism, repair mechanisms, immune response, and the vascular system, together with exogenous factors including brain traumata and overall life style [8-12]. By far the strongest genetic risk factor for late onset AD is the ϵ4 allele of the apolipoproteinE (apoE) gene [13,14]. ApoE is a major lipoprotein in the brain and mediates transport of cholesterol and other lipids between neurons and glial cells [15,16]. However, whether altered lipid transport in the brain via apoE contributes to the pathogenesis of AD is not well understood and requires more research [15,17]. Importantly, apoE is also linked to the metabolism of Aβ by affecting its aggregation in and clearance from the brain [18].

The importance of lipid metabolism in the brain is, however, evident from a number of other severe neurodegenerative diseases, caused by impaired degradation and transport of membrane lipids. These diseases are commonly dubbed as lysosomal lipid storage disorders (LLSDs) and characterized by strong accumulation of different lipids in endolysosomal compartments, in particular cholesterol and sphingolipids. Commonly, LLSDs are caused by loss of function mutations in genes encoding lipid catabolic proteins, including enzymes, lipid activator proteins or lipid transporters. Most of these diseases include neurological symptoms and show similarities at the cytopathological level to AD [8,19]. In the last years, several molecular mechanisms have been identified that connect membrane lipids to the metabolism of AD related proteins, in particular Aβ generation and aggregation. Studies so far have focused on the role of cholesterol and sphingolipids that are highly enriched in detergent-resistant membrane microdomains, also called lipid rafts. In turn, secretases, APP and its derivatives also appear to influence the membrane lipid composition by altering the activity of lipid metabolic enzymes and subcellular trafficking. These findings suggest a close interaction of metabolic pathways related to APP and membrane lipids. Thus, alterations in secretase activities as well as dysregulation of lipid metabolic enzymes might underlie the initiation and progression of AD pathogenesis.

### Secretases and cellular metabolism of APP

**Figure 1:**
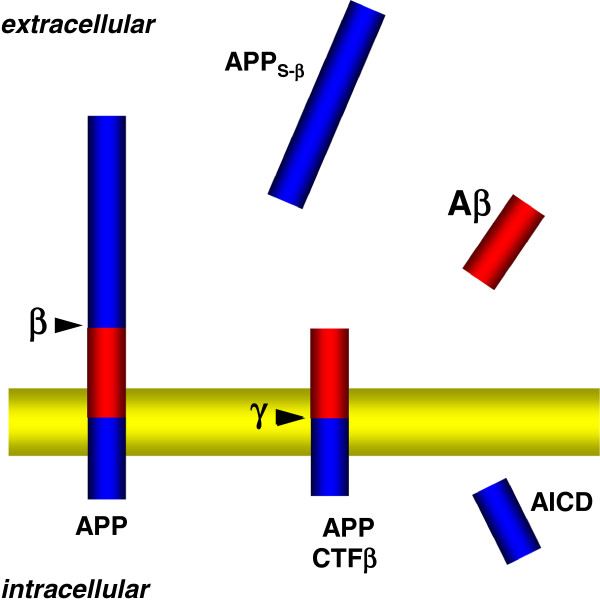
**Proteolytic generation of Aβ.** APP is cleaved by β-secretase resulting in the generation of membrane-tethered CTF-β and secretion of APP_S-β_. The CTFβ contains the full Aβ domain and subsequent cleavage by γ-secretase liberates Aβ into the extracellular milieu and the APP intracellular domain (AICD) into the cytosol.

APP is a type I membrane protein and follows the conventional secretory pathway from the endoplasmic reticulum (ER) to the plasma membrane. During this process, APP undergoes several co-and post-translational modifications, including N-and O-glycosylation, tyrosine sulphation, and phosphorylation [20,21]. Already on the way to the cell surface, APP can undergo endoproteolytic processing by secretases. The cleavage of full-length APP by α-or β-secretases within or at the N-terminus of the Aβ domain generates the soluble variants APPs-α and APPs-β, respectively, that can be secreted into the extracellular milieu (Figure 1). The remaining C-terminal fragments (CTFs) are still tethered to cellular membranes via their transmembrane domain. The CTFs generated by α-(CTFα) or β-secretase (CTFβ) become substrates for γ-secretase that cleaves within the transmembrane domains resulting in the secretion of the small peptides p3 and Aβ, respectively, and the liberation of the APP intracellular domain (AICD) into the cytosol (Figure 1).

Like APP, all secretases are integral membrane proteins. While α-and β-secretases have also type I topology, γ-secretase is a polytopic protein complex consisting of four individual components essential for the efficient cleavage of protein substrates. The PS proteins are the catalytically active components within this complex. The additional proteins anterior pharynx defective (aph) 1, presenilin enhancer (pen) 2, and nicastrin exert functions in assembly, subcellular transport, and substrate recognition [22-25]. All three secretases cleave a large number of additional substrates beside APP, and thus, exert multiple biological functions, including regulation of development, differentiation and proliferation [26-29].

It is important to note that in addition to the proteolytic processing by α-, β-, and γ-secretases, APP and its derivatives can also be metabolized in additional pathways including degradation by the proteasome and within lysosomal compartments [30-34]. Extracellular and luminal Aβ can also be degraded by certain members of the metallo-, serine-, aspartyl-, cysteine-protease families [35-38].

### Membrane lipids in the regulation of AD associated proteins

Apart of adipose tissue the mammalian brain contains the highest amount of lipids in the body. Although the central nervous system represents only 2% of the whole body mass it contains about 25% of the total unesterified body cholesterol and is the cholesterol richest organ of the body [39]. Free brain cholesterol is associated with the plasma membranes of neurons and glial cells on the one hand and with the specialized membranes of myelin on the other hand. In addition to cholesterol these membranes also contain complex sphingolipids such as glycosphingolipids, of which especially the sialic acid-containing gangliosides are particularly abundant and expressed in characteristic profiles in different neural cell types [40]. There is convincing evidence on the role of lipids as modulators of proteins involved in AD (see below), however, reports on changes in lipid contents in brains, cerebrospinal fluid and plasma of AD patients appear inconclusive. Changes of sphingolipids and cholesterol during neurodegeneration have been extensively reviewed recently and thus, will not be further described here [8,16,41-43]. Phospholipid levels were reported to be decreased especially in brain regions highly affected in AD [44]. Phospholipid changes in the brain, the cerebrospinal fluid and also in plasma at different stages of AD have also been recently reviewed [45].

#### Cholesterol and isoprenoids

APP and the secretases are embedded in the lipid bilayer of cellular membranes [17,46-48]. Thus, it is not surprising that the membrane lipid composition affects the proteolytic processing of APP. Early studies showed that, Aβ together with full-length APP, APP-CTFs, and PS1 were associated with detergent-resistant membrane microdomains (DRM) also called lipid rafts, [49-51]. Initial studies with cultured cells showed that inhibition of cholesterol biosynthesis by statins or cholesterol extraction from cellular membranes with β-cyclodextrin decreased Aβ production [52,53]. Notably, slight decreases in membrane cholesterol could also promote the secretion of Aβ [54]. Cholesterol is enriched in and affects the dynamics of lipid rafts. Because APP and its derivatives together with secretases partially distribute to rafts, changes in rafts structure by altered cholesterol levels might affect the localization of APP and secretases in these microdomains [17,55-58]. Biochemical isolation of DRMs also revealed the presence of beta-site APP cleaving enzyme (BACE1) and γ-secretase proteins PS1 and PS2, aph-1, pen-2 and nicastrin, while the α-secretase ADAM10 is predominantly localized outside of DRMs [59,60]. Interestingly, full-length APP also mainly distributes to non-DRM fractions, while the CTFβ derived from β-secretase mediated cleavage of APP show higher association with DRMs [49,59]. A recent NMR study showed the specific interaction of APP-CTFβ with cholesterol in the Aβ domain [61], which might underlie the enrichment of CTFβ in cholesterol-rich rafts. Moreover, the binding of cholesterol to CTFβ might directly affect its processing by γ-secretase. Interestingly, cholesterol-derived steroid hormones were recently shown to directly modulate γ-secretase processivity resulting in altered production of Aβ length variants, and it was proposed that a potential interaction of the carboxyl group of acidic steroids with a positively charged lysine residue in APP-CTFβ is responsible for the reduced production of Aβ42 [62]. However, these steroids might also affect γ-secretase activity via modulation of lipid raft composition.

The specific targeting of the β-secretase BACE1 to lipid rafts by addition of an GPI-anchor also increased Aβ production, suggesting that wild-type BACE1 is not quantitatively targeted to rafts under physiological conditions [63]. The association of BACE1 as well as of the γ-secretase components aph-1 and nicastrin with rafts might be dependent on their palmitoylation state [59]. However, further studies are required to understand the molecular mechanisms that regulate distribution of APP and secretases to lipid rafts and how this might affect Aβ generation.

The esterification rate of cholesterol can also affect the proteolytic processing of APP. Inhibition of Acyl-coenzyme A: cholesterol acyltransferase (ACAT1) decreases Aβ secretion in cellular models [64], and also strongly reduced plaque load in APP transgenic mice [65]. However, the molecular mechanisms underlying the beneficial effects of ACAT1 inhibitors *in vivo*, remain to be identified, as no hints for altered α-or β-secretory cleavage of APP have been found [65].

Cholesterol levels and transport might also affect the metabolism and aggregation of tau. Interestingly, human brains from NPC patients also revealed abundant neurofibrillary tangles very similar to that observed in AD brains, but no extracellular amyloid plaques [66-69]. NPC disease is mainly caused by mutations in NPC1 or NPC2 genes that encode late endosomal/lysosomal proteins involved in cholesterol transport and esterification. Thus, a primary defect in cholesterol transport in neurons might induce accumulation of tau independent of Aβ. In line with this notion, the deletion of NPC1 in mice leads to accumulation of free cholesterol and increased levels of hyperphosphorylated tau thereby resembling molecular changes of tau in AD. However, it is important to note that amyloidogenic CTFs of APP are increased in human and mouse NPC brains [70-72]. The exact molecular mechanisms underlying these observations remain to be determined in more detail. However, accumulating evidence indicates impairment of autophagy or lysosomal capacity in NPC cells which might contribute to the accumulation of APP-CTFs and tau, because both proteins can be degraded within autophagic and lysosomal pathways [8,32,71]. Also the activities of tau phosphorylating kinases, including microtubule associated protein kinases and cdk5 are upregulated in NPC cells [73,74]. Increased phosphorylation of endogenous tau was also observed in mice fed with high fat/cholesterol diet [75]. Moreover, high cholesterol diet also increased hyperphosphorylated tau and ongoing tau pathology in tau transgenic mice [76]. In turn, the deletion of the tau gene exacerbates the NPC phenotype in mice, suggesting that tau is not only degraded during autophagy, but also exerts important functions in this process, likely regulating transport and fusion of autophagic vesicles [77].

Isoprenoids that also derive from the cholesterol biosynthesis pathway can affect the transport and metabolism of APP as well as of tau [78-81]. The isoprenoids farnesylpyrophosphate and geranylgeranylpyrophosphate can be attached to certain proteins, including the small GTPases Rho that signal to the Rho-associated kinase (ROCK). The inhibition of HMG-CoA reductase by statins also decreases the biosynthesis of isoprenoids. This effect has indeed been shown to affect Rho-Rock signaling to increase α-secretory processing of APP in cultured cells, which might also affect Aβ generation [78]. The inhibition of >Rho-Rock signaling has also been shown to decrease the (hyper)phosphorylation of tau [79,80].

Epidemiologic studies indicate that statin intake could decrease the risk of developing AD [82-84]. However, a protective role of statins against AD could not be observed in other studies. Randomized controlled prospective trials with AD patients also showed inconclusive results ranging from beneficial to ineffective [17,83]. The use of different statins with different permeabilities for the blood brain barrier, different sample sizes and outcome measures could have contributed to these differing results. It is also unclear whether the potentially preventive effects of statins involve indeed lower cholesterol levels or also additional pleiotropic effects of these drugs. It will thus be important to further investigate the relative contribution of isoprenoid and cholesterol metabolic pathways to the potentially protective role of statins in AD pathogenesis [85,86]. It has been shown that statin treatment of cultured cells also promotes the degradation of Aβ by increasing the unconventional secretion of the insulin-degrading enzyme [87]. The statin depended effects were observed without changes in cellular cholesterol concentrations and could be attributed to impairment of protein farnesylation [87,88]. Thus, modulation of isoprenoid metabolism not only affects the generation, but also the clearance of Aβ.

#### Sphingolipids

Sphingolipids (SLs) are closely associated with cholesterol in lipid rafts [89]. The metabolism of SLs is closely associated with cell survival and cell death [90]. In particular, ceramide is a pro-apoptotic signaling molecule [91], and thus might be involved in different neurodegenerative diseases [92,93]. Here we focus on the molecular mechanisms underlying SL-dependent metabolism of APP.

Ceramide, the membrane anchor of SLs was shown to stabilize BACE1 and increase Aβ secretion in cultured cells [94]. In turn, the genetic or pharmacologic inhibition of SL biosynthesis decreased Aβ generation, likely involving decreased forward transport and maturation of APP in the secretory pathway [95-97]. SLs also appear to decrease the lysosomal degradation of APP thereby providing more substrate to secretases to increase the generation of soluble APP variants and Aβ [33,95,98]. However, contrasting results were observed in CHO cells with defective SL biosynthesis that rather secreted more Aβ42 [97]. Thus, lowering SL levels might affect the proteolytic processing of APP and Aβ generation by several mechanisms and effects might be dependent on the cell type and experimental conditions.

A potential role of ceramide in tau metabolism is also supported by a study in PC12 cells where ceramide analogs decreased the levels of tau [99]. However, addition of the ganglioside GM1 increased levels of tau and stabilized the microtubule network in neuroblastoma cells [100]. These effects were associated with redistribution of MAP2 and enhanced neurite outgrowth [100,101].

**Figure 2:**
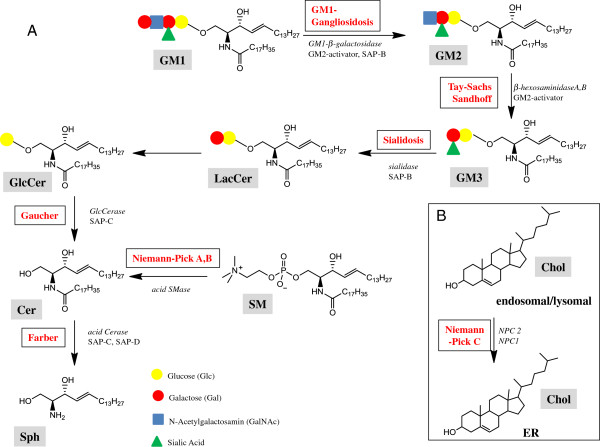
**Lipid degradation and lysosomal lipid storage diseases. A)** Sequential degradation pathways of selected (glyco)sphingolipids in which hydrolytic enzymes catalyzing SL degradation often need the assistance of an additional protein (GM2-activator or one of 3 saposins: SAP-B,-C,-D as indicated). **B)** Cholesterol storage in the late endosomal/lysosomal compartment due to mutated NPC1 or NPC2 proteins mediating its transport to post-lysosomal compartments (e.g. the ER). The names of respective diseases are indicated. Cer, Ceramide, Gal, D-galactose; GalNAc, N-Acetyl-D-galactosamine; Chol, cholesterol; Glc, D-glucose; GlcCer, glucosylceramide; LacCer, lactosylceramide; the terminology used for gangliosides GM1, GM2, GM3 is that of Svennerholm [106]; SM, sphingomyelin, Sph, sphingosine, Cerase, ceramidase; GlcCerase, Glucosylceramide-β-glucosidase; SMase, sphingomyelinase; SAP, sphingolipid activator protein, saposin. For detailed schemes on SL metabolism see [8].

A number of studies showed that accumulation of SLs increased levels of APP and secretion of Aβ [32,95,98]. This was also observed in cellular and mouse models with impaired degradation of SLs that therefore resemble human LLSDs, including Niemann-Pick type A and B, Tay-Sachs and Sandhoff disease (Figure 2) [32,72,102]. The accumulation of lipids can impair lysosomal function and thereby lower the capacity of cells to degrade APP and its derivatives [32,103]. The genetic deletion of GD3 synthase and thereby inhibition of the biosynthesis of b-series gangliosides reduced Aβ deposition and improved memory deficits in APP transgenic mice [104]. Mice with deleted GM2 synthase gene that lack GM1, but have increased expression of GM3 showed more complex changes in Aβ deposition [105]. Interestingly, these mice developed in addition to a slight increase in Aβ plaque load in the parenchyma, also prominent vascular amyloid angiopathy [105]. Thus, gangliosides might not only affect the general deposition, but also influence the region specific formation of Aβ aggregates.

Furthermore, sphingosine 1-phosphate (S1P) and certain other SLs can directly stimulate the activity of BACE1, independent of changes in the trafficking or stabilization of the protease in cells [107,108]. The exact mechanisms remain to be determined, but might involve electrostatic interactions of the lipid headgroups with the catalytic ectodomain of BACE1. This is further supported by a stimulatory effect of certain brain gangliosides on BACE1 variants lacking the tramsmembrane domain [108]. Note that S1P was also reported to promote tau phosphorylation via a calcium/calpain and cdk5 mediated mechanism [109].

SLs can also regulate the activity of purified γ-secretase [110]. The addition of exogenous SLs to purified γ-secretase complexes or to isolated cellular membranes not only increased overall activity but also changed the cleavage specificity of γ-secretase to elevate Aβ42/Aβ40 ratio [32,110,111].

Several mechanisms might underlie the effects of cholesterol and SLs on secretase activities. Membrane lipids could directly interact via their hydrophobic moieties with the transmembrane domains of BACE1, the subunits of the γ-secretase complex or of their substrate APP. Interactions with secretases or APP could also be mediated via polar headgroups of membrane lipids. For example, the ganglioside GM1 has been shown to directly bind to the N-terminal domain of full-length and secreted APP thus changing its conformation. Because other SLs did not interact with the APP ectodomain, the glycomoiety of GM1 might determine this interaction. Thus, subcellular transport and proteolytic processing of APP might also be modulated by direct interaction with the head groups of SLs [112].

In addition, there is convincing experimental evidence for the role of membrane lipids not only for the generation of Aβ (see above), but also for their particular role in shifting its conformation from helix to beta-sheet rich structures. Particularly raft-associated ganglioside GM1, which is especially abundant in the hippocampus was shown to promote conformational changes of Aβ [113-115]. The initial crucial finding was the unique GM1-bound form of Aβ, the so called GAβ [113]. Studies with a specific anti-GAβ antibody convincingly argued in favor of an essential role of raft-associated-gangliosides in the polymerization of Aβ in AD [116]. GAβ was detected not only in human AD, but also in aged monkey brains [117]. In addition, GAβ formation could be correlated with presynaptic terminal-specific Aβ deposition, being favored by known AD risk factors like aging and expression of apoE4 [118,119]. Notably, accumulation of GAβ occurred exclusively in subcellular structures of the endocytic pathway, the main site of Aβ generation [120]. Aβ can also interact with GM3. It has has been proposed that binding of Aβ to GM3 inhibits GD3 synthase, thereby changing cellular ganglioside profiles [121].

#### Phosphoglycerides

Most research related to the role of lipids in APP processing and Aβ generation has been focused on cholesterol and sphingolipids. However, phosphoglycerides (PGs) are the main constituents of biological membranes. PGs not only exert structural functions, but also are important for cellular signal transduction. PGs are metabolized to produce potent signaling molecules, including inositol-1,4,5-trisphosphate, diacylglycerol, and phosphatidic acid [122-124]. These metabolites regulate multiple pathways in cells by controlling Ca^2+^ signaling or kinase and phosphatase activities that are also implicated in the complex regulation of APP metabolism. However, the pleitropic roles of PGs in cellular signaling complicate the analysis of specific effects of individual lipids on APP processing in cellular and in vivo models [58].

In vitro systems with liposomes or purified cellular membranes, demonstrated direct effects of PGs on the activites of BACE1 and γ-secretase. Increasing the concentration of anionic glycerophospholipids stimulated BACE1 activity in reconstituted liposomes [108]. Under these experimental conditions, a contribution of intracellular signaling pathways could be ruled out. Thus, PGs might directly affect enzyme activity, likely involving interaction of lipid head groups with the catalytic domain of BACE1.

A systematic analysis on the influence of membrane thickness revealed that C18 and C20 fatty acids in phosphatidylcholine potently stimulated purified γ-secretase as compared to phosphatidylcholine with shorter C16 and C14 or longer C22 and C24 fatty acids. Notably, increased membrane thickness decreased the ratio of Aβ42 to total Aβ [125]. Together these data indicate that membrane thickness not only affects the overall activity, but also the cleavage specificity of γ-secretase. As the chain length of fatty acids in membrane lipids is also affecting membrane fluidity, these effects might reflect changes in membrane thickness, but also in the lateral mobility of enzymes and protein substrates. However, as membrane thickness differs between distinct subcellular compartments, these characteristics of different membrane systems could strongly affect the generation of different Aβ species. Inhibitory effects on purified γ-secretase were observed for phosphoinosites [126] and plasmalogens [127]. From the phosphatidylinositols tested, phosphatidylinositol(4,5)bisphosphate was most potent in γ-secretase inhibition, while phosphatidylinositol and phosphatidylinositol(3,4,5)trisphosphate had negligible effects.

### AD associated proteins and the metabolism of membrane lipids

As described so far, membrane lipids exert multiple effects on APP processing. Interestingly, recent studies also revealed a regulatory role of APP and its derivatives as well as of secretases in cellular lipid metabolism [8,47].

**Figure 3:**
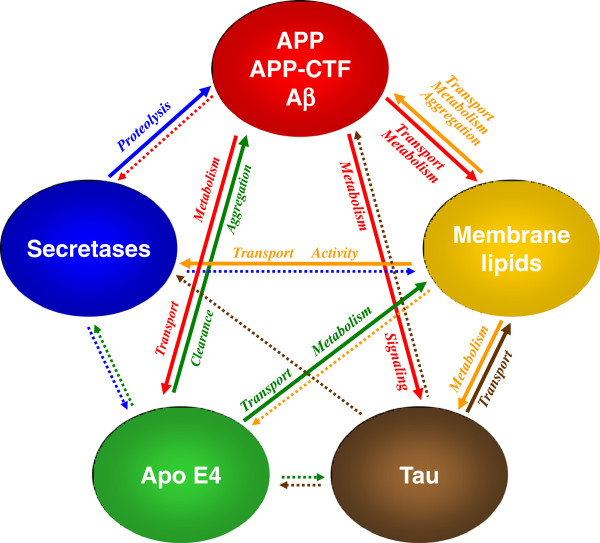
**Cross-talk of membrane lipids and Alzheimer-associated proteins.** Alterations in membrane lipid composition affect secretase activities, thereby modulating APP processing and generation of Aβ. Alternatively, membrane lipids can directly interact with Aβ and modulate its aggregation. In addition, membrane lipids impair the metabolism of tau. Thus, both neuropathological hallmarks of AD could be triggered by age-dependent changes in lipid metabolism. Conversely, membrane lipid composition is affected by APP and its derivatives Aβ and CTFβ, which were shown to modulate lipid metabolic enzymes and directly bind membrane lipids including cholesterol and gangliosides. Tau also affects membrane lipid composition, likely via regulation of vesicular transport. ApoE as a major lipoprotein in the brain could also affect lipid composition, but also Aβ clearance and aggregation. Solid arrows indicate a direct interaction of the respective components whereas dotted arrows indicate potential modulations by yet undefined mechanisms. See text for further details.

APP and its derivatives generated by γ-secretase can contribute to the regulation of lipid metabolic pathways (Figure 3). Aβ itself can alter the activity of enzymes involved in sphingolipid and cholesterol metabolism. Aβ42 increased the activity of neutral SMase and thereby decreased SM levels in cultured cells, while Aβ40 inhibited HMG-CoA reductase and lead to decreased cholesterol biosynthesis [128]. Alternatively, Aβ-dependent increases in ceramide and cholesterol levels might be mediated by membrane-associated oxidative stress [129-131]. In line with the effect of FAD associated mutations in PS proteins on Aβ42/40 ratios, expression of FAD mutant PS1 increased cholesterol levels, but decreased SM levels. Increased cholesterol levels were also observed in cells from PS KO mice and in brains of mice expressing FAD-mutant PS1 [132,133]. However, the studies proposed alternative mechanisms underlying the changes in cellular cholesterol levels. The γ-secretase cleavage product AICD could act as a transcriptional regulator of the LDL receptor related protein 1(LRP1). As AICD negatively regulates LRP1 transcription, LRP1 protein expression was increased in PS1 deficient cells where AICD production by γ-secretase is inhibited. Thus, extracellular cholesterol complexed with apoE could be internalized more efficiently in PS deficient cells thereby increasing cellular cholesterol levels [132]. However, own work demonstrated that the uptake of lipoproteins is rather decreased in PS deficient FAD mutant cells and mouse brain [133]. The deficit in the internalization of extracellular cholesterol in turn upregulated cholesterol biosynthetic genes including SREBP2 and CYP51, resulting in an overproduction of cholesterol [133]. A recent study demonstrated that a significant pool of PS protein is localized in membrane-associated mitochondria (MAM), sites with close contacts of mitochondrial and ER membranes [134,135]. MAM structures were increased in PS KO or PS1 FAD mutant cells, suggesting that PS proteins and associated γ-secretase activity negatively regulated MAM contacts. PS deficient cells also showed increased biosynthesis of cholesterol [135]. Interestingly, MAMs appear to be important for the generation of cholesterol esters and their storage in lipid droplets. In line with an increased number and size of MAMs, cholesterol esters and lipid droplets were found to be significantly increased in PS deficient cells. While further studies are required to dissect the molecular pathways, it is evident that γ-secretase activity is closely related to cellular cholesterol metabolism.

γ-Secretase has also been linked to phosphatidylinositol metabolism [136]. In cells expressing PS1 FAD mutants, the level of Aβ42 showed inverse correlation to phosphatidylinositol(4,5)bisphosphate. This effect was attributed to increased degradation of this phosphatidylinositol by phospholipase C to inositol-1,4,5-trisphosphate and diacylglycerol [136]. However, whether phospholipase C activity is directly affected by Aβ in these models or other mechanisms are also involved remains to be determined. Most studies so far have been carried out in non-neuronal cell lines. Thus, it will be important to investigate the functional role of AD associated proteins in lipid metabolism in neurons. A recent study revealed that the pharmacologic inhibition of γ-secretase selectively increased ganglioside concentration in neuritic terminals of differentiated PC12 cells [137]. Whether impaired metabolism of APP was involved in these effects remained unclear. A direct involvement of APP in neuronal lipid metabolism came from studies with primary rat cortical neurons [138]. Overexpression of human APP decreased cholesterol de novo synthesis associated with decreased expression of HMG-CoA reductase and SREBP1, while down-regulation of endogenous APP expression had opposite effects resulting in increased cholesterol synthesis. These effects were attributed to a direct interaction of APP with SREBP1 and negative regulation of SREBP1 target genes. Surprisingly, the interaction of both proteins and regulation of cholesterol biosynthesis was not observed in astrocytes, suggesting a neuron specific role of APP in cholesterol metabolism.

The role of tau in the regulation of lipid metabolism is much less characterized. In human AD brains, tangle bearing neurons showed increased immunoreactivity for the lipid raft associated protein flotilin-1 in lysosomes, suggesting accumulation of cholesterol and sphingolipids in these compartments [139]. Hyperphosphorylated tau has also been shown to be associated with lipid rafts in APP transgenic mice. In addition, small amounts of cholesterol, sphingolipids and phosphatidylcholine were also found in purified paired helical filaments [140]. Given its role in subcellular transport of vesicles along microtubules, it is likely that the effects of tau on membrane lipids involve altered vesicular transport of lipids and/or [141] lipid metabolizing proteins.

## Conclusion

AD is associated with complex changes in the metabolism of membrane lipids. However, the available data suggest that changes in cellular lipid metabolism could not only be a consequence of, but also trigger or at least promote, AD pathogenesis (Figure 3). Thus, impaired homeostasis of membrane lipid composition could be an initial event in the etiology of AD. One of the earliest cytopathological changes in AD is an increased number and size of endolysosomal compartments, suggesting impairment of the lysosomal clearance capacity [71,141]. These changes are highly similar to LLSDs, were the primary defect causes strong accumulation of membrane lipids in endolysosomal compartments [8,142]. Notably, characteristic AD related changes, including increased levels of Aβ and amyloidogenic fragments of APP, hyperphosphorylated tau and neurofibrillary tangles together with neuroinflammation were also observed in mouse models as well as human brain samples of certain LLSDs [143,144].

Taken together, the targeting of lipid metabolism could represent a promising strategy in AD therapy and prevention. Moreover, lipids could also be explored further for their potential as biomarkers for early diagnosis or even prognosis of AD. Thus, it will be interesting to unravel the complex interplay of lipid and protein metabolism and their relevance in neurodegenerative diseases in the future.

## Abbreviations

ACAT: Acyl-coenzyme; A: Cholesterol acyltransferase; AD: Alzheimer’s disease; AICD: APP intracellular domain; Aβ: Amyloid β-peptide; APP: Amyloid precursor protein; apoE: ApolipoproteinE; BACE1: Beta-site APP cleaving enzyme; CTF: C-terminal fragment; DRM: Detergent-resistant membrane microdomain; ER: Endoplasmic reticulum; FAD: Familial Alzheimer’s disease; GAβ: GM1-ganglioside-bound-Aβ; LLSD: Lysosomal lipid storage disorder; NFT: Neurofibrillary tangles; NPC: Niemann Pick disease type C; pen: Presenilin enhancer; PGs: Phosphoglycerides; PHF: Paired helical filaments; PS: Presenilin; S1P: Sphingosine 1-phosphate; SL: Sphingolipid; SM: Sphingomyelin.

## Competing interests

The authors declare that they have no competing interests.

## Authors’ contribution

JW and GvE-D wrote the manuscript. Both authors read and approved the final manuscript.
